# *O*-GlcNAcylation and Phosphorylation Crosstalk in Vascular Smooth Muscle Cells: Cellular and Therapeutic Significance in Cardiac and Vascular Pathologies

**DOI:** 10.3390/ijms26073303

**Published:** 2025-04-02

**Authors:** Israel O. Bolanle, Timothy M. Palmer

**Affiliations:** Biomedical Institute for Multimorbidity, Centre for Biomedicine, Hull York Medical School, University of Hull, Hull HU6 7RX, UK; olapeju.bolanle@hyms.ac.uk

**Keywords:** *O*-GlcNAcylation, phosphorylation, crosstalk, vascular smooth muscle cells

## Abstract

More than 400 different types of post-translational modifications (PTMs), including *O*-GlcNAcylation and phosphorylation, combine to co-ordinate almost all aspects of protein function. Often, these PTMs overlap and the specific relationship between *O*-GlcNAcylation and phosphorylation has drawn much attention. In the last decade, the significance of this dynamic crosstalk has been linked to several chronic pathologies of cardiovascular origin. However, very little is known about the pathophysiological significance of this crosstalk for vascular smooth muscle cell dysfunction in cardiovascular disease. *O*-GlcNAcylation occurs on serine and threonine residues which are also targets for phosphorylation. A growing body of research has now emerged linking altered vascular integrity and homeostasis with highly regulated crosstalk between these PTMs. Additionally, a significant body of evidence indicates that *O*-GlcNAcylation is an important contributor to the pathogenesis of neointimal hyperplasia and vascular restenosis responsible for long-term vein graft failure. In this review, we evaluate the significance of this dynamic crosstalk and its role in cardiovascular pathologies, and the prospects of identifying possible targets for more effective therapeutic interventions.

## 1. Introduction

*O*-GlcNAcylation, a dynamic, reversible post-translational modification (PTM) that involves adding the monosaccharide β-D-*N*-acetylglucosamine (*O*-GlcNAc) to serine or threonine residues on target proteins, has been linked to the regulation of multiple cellular functions and disease pathologies [1]. Since its discovery by Hart and Torres in the 1980s [2], a growing body of evidence has described its diverse roles in the development of multiple cardiovascular diseases [3,4,5,6,7,8,9,10]. Additional research findings have shown that *O*-GlcNAcylation targets a number of key proteins essential for maintaining cardiovascular homeostasis [11,12]. Examples include sarco/endoplasmic reticulum calcium (Ca^2+^)-ATPase (SERCA2), phospholipase C-beta 1 (PLC-β1), isoforms of protein kinase C (PKC) such as PKC-β1, PKC-δ, and PKC-ε, endothelial nitric oxide synthase (eNOS), and phosphatidylinositol 3-kinase (PI3K) [13,14,15]. On the other hand, phosphorylation, a modification that involves a phosphoryl group being primarily covalently attached to the hydroxyl groups of specific serine, threonine, and tyrosine residues, is undoubtedly the most studied PTM [16]. It is estimated that approximately 30% of the human proteome is phosphorylated at any given time, which can alter target protein localisation, and activity [16,17]. The regulation of several cardiovascular functions, including myocardial and vascular smooth muscle contraction, is closely linked to the phosphorylation status of critical protein targets [18]. Well-studied examples include cAMP-dependent protein kinase (PKA)-mediated regulation of myocardial contraction and Ca^2+^-calmodulin protein kinase (Ca^2+^/CAM-PK) [19,20]. Other important examples include cGMP-dependent protein kinase (PKG), tyrosine protein kinases (PTKs), and several isoforms of PKC including PKC-β1, PKC-δ, PKC-ε, PKC-η, and PKC-γ [21,22,23,24]. Additional cardiovascular system-regulating proteins that have been found to be phosphorylated include c-jun N-terminal protein kinases (JNKs) and mitogen-activated protein kinases (MAPKs), which include extracellularly regulated kinases (ERKs) [25,26,27,28].

Proteomic data have indicated that there are approximately 80,000 identified sites on target proteins where PTMs occur [29,30]. However, serine and threonine residues are specifically known to be the main locations for *O*-GlcNAcylation and phosphorylation [31,32]. Hence, it is not surprising that these two PTMs have been shown to crosstalk with each other to regulate protein function. In recent years, several studies have focused on elucidating the potential significance of this crosstalk in cardiovascular diseases [29,33,34,35,36]. However, the conclusions have not been clear cut, with data demonstrating two opposing outcomes; promotion (friend) or inhibition (foe) of one another [29,36]. It has also been observed that the outcomes can be cell-specific [29,36]. However, the significance of such crosstalk on target proteins in vascular smooth muscle cells (VSMCs) is unknown. VSMCs are key cells that play important roles not only in the regulation of vascular tone and blood pressure, but also in the pathogenesis of atherosclerosis and hypertension [37]. VSMCs have also been implicated in the vascular dysfunction responsible for vascular restenosis and vein graft failure [7]. Thus, our aim in this review is to highlight the cellular significance of this dynamic crosstalk in VSMCs with the goal of identifying viable targets for therapeutic interventions.

## 2. *O*-GlcNAcylation and Phosphorylation Crosstalk

**Figure 1:**
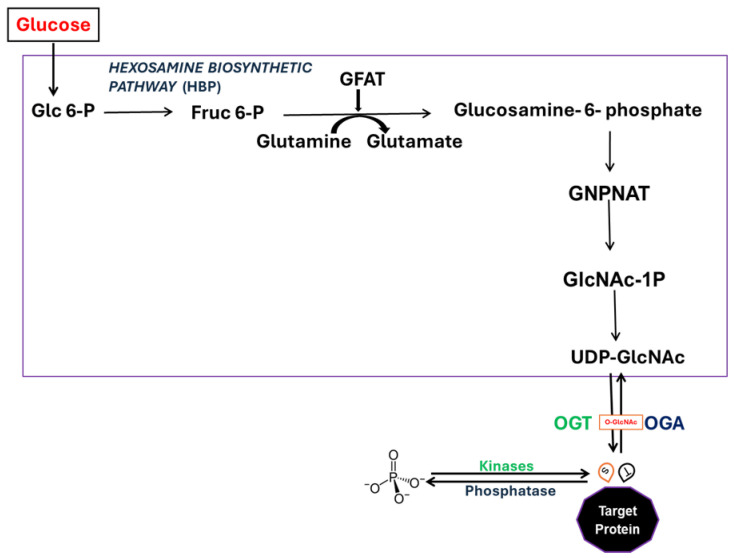
Schematic of the hexosamine biosynthetic pathway. Glucose is converted to fructose-6P (fructose-6-phosphate) once it enters the HBP. Next, glutamine-fructose-6P amidotransferase 1 (GFAT), the HBP’s rate-limiting enzyme, adds an amino group from glutamine to fructose-6-phosphate to create glucosamine-6-phosphate (GlcN-6-P). Following this, in the presence of acetyl-CoA, glucosamine-phosphate *N*-acetyltransferase (GNPNAT, EMeg32) quickly acetylates GlcN-6P to form *N*-acetylglucosamine-6-phosphate (GlcNAc-6P). GlcNAc phosphomutase (PGM3/AGM1) then isomerises this compound to produce *N*-acetylglucosamine-1-phosphate (GlcNAc-1P). This is then added to the sugar by UDP-*N*-acetylhexosamine pyrophosphorylase 1 (UAP/AGX1) to yield UDP-GlcNAc which is the amino sugar substrate for protein *O*-GlcNAcylation. Protein *O*-GlcNAcylation is a nutrient- and stress-responsive PTM controlled by two enzymes, *O*-GlcNAc transferase (OGT) and *O*-GlcNAcase (OGA). While OGT catalyses the attachment of the *O*-GlcNAc moiety from a UDP-GlcNAc substrate to target proteins, OGA reverses this process [47]. *O*-GlcNAcylation occurs on serine and threonine residues of target proteins, sites that may also be targets for phosphorylation by Ser/Thr-directed protein kinases. Glc-6P (Glucose-6-phosphate), Fruc-6P (fructose-6-phosphate), GFAT (glutamine:fructose-6-phosphate amidotransferase), GNA1/GNPNAT1 (glucosamine-6-phosphate *N*-acetyltransferase), GlcNAc-1P (*N*-acetylglucosamine-1-phosphate), PGM3/AGM1 (phosphoglucomutase), UDP-GlcNAc (uridine diphosphate-*N*-acetylglucosamine), S (serine), and T (threonine).

We have extensively described protein *O*-GlcNAcylation and its biochemistry in our previous reviews (Figure 1; [5,7]), and Chatham and Patel [38] have provided a detailed account of its role in cardiovascular health and disease. *O*-GlcNAcylation has been demonstrated to regulate a number of cellular processes, including gene transcription [39], neuronal function [40], epigenetic regulation [41], cell signalling dynamics [42], modulation of protein–protein interactions [43], and the response to external stressors [44]. Phosphorylation is already possible for nearly all *O*-GlcNAcylated proteins [31,42]. Extensive mapping of *O*-GlcNAc sites has further revealed that phosphorylation can occur at the same sites [45]. Additionally, developments in mass spectrometric analysis techniques to detect *O*-GlcNAcylated sites coupled with conventional metal ion affinity techniques to analyse phosphorylated proteins has also revealed that there is extensive dynamic crosstalk between *O*-GlcNAcylation and phosphorylation [34,46]. While this has attracted a lot of interest with the aim of clarifying its potential therapeutic relevance, the characterisation has not yet yielded a significant breakthrough because of the inherent complexity. However, existing findings suggest *O*-GlcNAcylation and phosphorylation have a “friend or foe” relationship which depends not only on the target protein but also on the sites and cell types [31].

### 2.1. *O*-GlcNAcylation and Phosphorylation Operating as Friends

Exposure of rat aortic VSMCs to a high pathophysiological glucose concentration (20 mM), known to enhance OGT-mediated *O*-GlcNAcylation [5], resulted in upregulated protein *O*-GlcNAcylation and increase in the activity of several PKC isoforms, such as PKC-α, PKC-β, PKC-δ, PKC-ε, and PKC-γ, which are typically activated by phosphorylation [48]. This observation suggests a so-called friendly relationship and supports earlier findings that a reduced intracellular response of VSMCs to vasoactive hormones is caused by glucose-mediated activation of PKC rather than by osmolality [49]. In the study, William and Schrier demonstrated that glucose-induced PKC activation decreases the angiotensin Il-mediated calcium signal in VSMCs [49]. These observations identify one important aspect of the significance of *O*-GlcNAcylation and phosphorylation crosstalk in the vasculature.

### 2.2. *O*-GlcNAcylation and Phosphorylation Operating as Foes

It has been demonstrated that activation of protein kinase A (PKA) or PKC in cerebellar neurons of NMRI mice downregulated *O*-GlcNAcylation and selective inhibitors of these kinases caused upregulation of *O*-GlcNAcylation [50]. In the study [50], following treatment with the inhibitors (bis-indoylmaleimide to inhibit PKC and KT5720 to inhibit PKA), *O*-GlcNAc levels were quantified by the enzyme-linked immunosorbent assay using MUD50, a mouse monoclonal antibody originally raised against glycoproteins from *Dictyostelium* known to be specific for *O*-GlcNAc-modified peptides [51]. Furthermore, it is suggested that *O*-GlcNAcylation’s competitive interaction with phosphorylation contributes to its role in signal transmission [34]. For example, *O*-phosphorylation on the C-terminal domain of RNA polymerase II [52], Thr-58 of c-Myc [53], Ser-16 of murine estrogen receptor-β [54], and Ser-1177 of endothelial nitric-oxide synthase (eNOS) [55], is reciprocal to *O*-GlcNAc. An additional study has also demonstrated that in several neuroblastoma cell lines, okadaic acid, an inhibitor of protein phosphatases PP1 and PP2A, globally elevated protein phosphorylation levels, which in turn resulted in decreased *O*-GlcNAcylation [56]. On the other hand, O-(2-acetamido-2-deoxy-d-glucopyranosylidene)-amino-N-phenylcarbamate (PUGNAc), a competitive OGA inhibitor, caused globally elevated *O*-GlcNAc levels, which resulted in insulin resistance in adipocytes and decreased phosphorylation-induced activation of Akt [57].

In a recent study by Umapathi et al. [58], increased *O*-GlcNAcylation was observed in the hearts of OGT transgenic mice, and the mice developed ventricular arrhythmias, severe dilated cardiomyopathy, and premature death. On the other hand, OGA transgenic hearts had reduced *O*-GlcNAcylation and were less susceptible to pathological stress caused by pressure overload, showing reduced pathological hypertrophy and attenuated myocardial *O*-GlcNAcylation levels following stress. This study [58] suggests that *O*-GlcNAcylation promotes cardiomyopathy and enhanced OGA activity protects against pathologic remodelling and heart failure brought on by pressure overload. Additionally, Clark et al. [59] have shown that sustained hyperglycaemia-induced increased *O*-GlcNAcylation caused a prolonged calcium transient decays in rat cardiomyocytes and lowers the expression of SERCA2a, a crucial sarcoplasmic calcium ATPase. They suggested that this observation is linked to hyper-*O*-GlcNAcylation of the Sp1 transcription factor and further showed that adenovirus-mediated expression of OGA could ameliorate this effect [59]. Further to these findings, Hu et al. [60] demonstrated that adenovirus-mediated overexpression of OGA (AdOGA) improved contractile function in NIH Swiss mice diabetic heart. Further investigation showed that treatment of diabetic heart with AdOGA caused increased level of phosphorylation, resulting in a 40% increase in active SERCA2a expression and a two-fold increase in the level of phosphorylated phospholamban protein [60]. Taken together, these findings suggest that upregulation of *O*-GlcNAcylation is detrimental to cardiac function and modulating this by lowering cellular *O*-GlcNAcylation improves diabetic heart function and calcium handling [60,61].

Furthermore, when phosphorylation of glycogen synthase kinase-3 (GSK-3), a single cytoplasmic serine/threonine protein kinase, is inhibited with lithium in COS7 cells, there was increased *O*-GlcNAcylation of at least 45 proteins including cytoskeletal proteins, heat shock proteins, RNA processing enzymes, transcription factors, ribosomal proteins, and metabolic enzymes [34]. GSK-3 regulates several cellular processes including cell proliferation, stem cell renewal, apoptosis and development. GSK-3 plays very important roles in the heart to control its development through the formation of heart and cardiomyocyte proliferation, and it is also known to be one of the key proteins regulating heart fibrosis and hypertrophy [62]. Also, Leney et al. performed an extensive elucidation of this crosstalk in A549 and HeLa cells [63], demonstrating that phosphorylation at specific sites hampers *O*-GlcNAcylation. For instance, Thr344 phosphorylation was found to be antagonistic to Ser347 *O*-GlcNAcylation in CK2 [63]. Additionally, it has been demonstrated that OGT overexpression significantly decreases phosphorylation of kinases crucial for cell division, including polo kinase, aurora kinase A, and cyclin-dependent protein kinase 1 [35,64].

Recent findings by Zhu et al. showed that activation of ATM- and Rad3-related (ATR) signalling by Forkhead box protein P1 (FOXP1) is repressed by FOXP1 *O*-GlcNAcylation [65]. ATR signalling is crucial for detecting and reacting to replication stress; hence, any malfunctions may affect the survival and function of cells. Complete kinase activity of ATR is only exhibited upon phosphorylation and in this study [65], Zhu et al. identified FOXP1 as an ATR-interacting protein and a regulator in promoting ATR activation in HEK293T cells when phosphorylated by checkpoint kinase 1 (CHK1). CHK1 is a major kinase that modulates cell cycle checkpoint activation and DNA repair [66]. However, Zhu et al. discovered that OGT-mediated FOXP1 *O*-GlcNAcylation compromised ATR activation by repressing its interaction with FOXP1 [65]. They further demonstrated that CHK1-mediated FOXP1 phosphorylation at S396 in HEK293T cells antagonises its *O*-GlcNAcylation [65].

Additionally, Wang et al. conducted an extensive quantitative analysis on 711 phosphopeptides using iTRAQ labelling, sequential phospho-residue enrichment and high throughput mass spectrometric analyses to ascertain the impact of globally elevated *O*-GlcNAcylation on site-specific phosphorylation dynamics [46]. They discovered that enhanced *O*-GlcNAcylation levels due to OGA inhibition caused enhanced and decreased phosphorylation at 148 and 280 sites, respectively [46]. Two potential mechanisms for this crosstalk include enzymatically dependent regulatory mechanisms and direct or steric competition [67]. For example, reduced phosphorylation of eNOS at Ser1177 (eNOS-Ser1177) and lower eNOS activity have been linked to enhanced *O*-GlcNAc modification of eNOS on the same residue [55]. This is an example of direct competition occurring at the same site, whereas in other proteins the competition could occur at either proximal or more distant sites [46].

**Figure 2:**
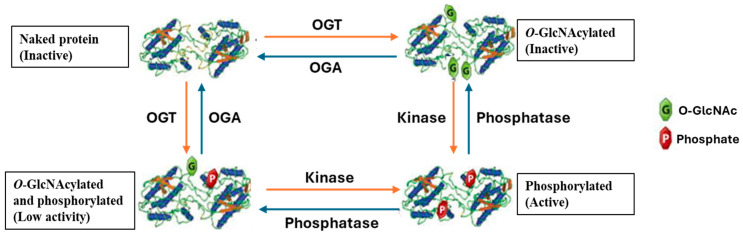
A schematic of how *O*-GlcNAcylation and phosphorylation crosstalk regulates calmodulin kinase IV (CaMKIV) activity. *O*-GlcNAcylated and -phosphorylated sites are demonstrated using the CaMKIV structure. In the inactive state, *O*-GlcNAcylation of the naked protein prevents proximal activating phosphorylation due to *O*-GlcNAc occupancy of residue sites, which makes the protein dormant. Upon activation, OGA hydrolyses the *O*-GlcNAc residue, making the phosphorylation sites available for kinase activation. However, there is usually reduced activity when there is shared occupancy of the sites or when phosphorylation of the sites is incomplete. OGT (*O*-GlcNAc transferase) and OGA (*O*-GlcNAcase).

Aside their crosstalk at the site occupancy level, *O*-GlcNAcylation and phosphorylation alter the enzymes that regulate one another’s cycling on polypeptides in a dynamic manner [35,68]. OGT and OGA are frequently found within multi-protein complexes that contain protein phosphatases and kinases [69,70,71]. For example, OGT is linked to protein phosphatase 1 catalytic subunits suggesting that the same enzyme complex can not only add *O*-GlcNAc but also remove phosphate from Ser/Thr residues on target proteins [71]. In addition to being modified by *O*-GlcNAc, a growing number of kinases are also known to be controlled by the sugar [72,73,74]. For example, calcium/calmodulin-dependent protein kinase IV (CaMKIV), an essential Ser/Thr-directed protein kinase that controls VSMC proliferation is also involved in the phosphorylation and activation of transcription factors such as Akt, mTOR, and S6K [75,76]. The phosphorylation of these transcriptional factors is predominantly responsible for the proliferative effect of CaMKIV [76]. For example, in various types of cancer (e.g., hepatocellular carcinoma, breast cancer, neuroblastoma, and prostate cancer), activation of mTOR/S6K signalling by CaMKIV results in translocation of S6K into the nucleus where it interacts with RORγt to cause transcriptional activation of IL-17-producing Th17 cells [76]. Th17 cells has been linked to the pathogenesis of several chronic inflammatory diseases such as atherosclerosis [77]. CaMKIV has many residues in its ATP-binding pocket and *O*-GlcNAcylation occurs at or near its activating phosphorylation site [78]. Dias et al., in this study [78], identified five *O*-GlcNAc sites on CaMKIV (Thr-57/Ser-58, Ser-137, Ser-189, Ser-344/Ser-345, and Ser-356) and evaluated their effect on identified phosphorylated sites (Ser-12/Ser-13, Thr200, and Ser 336) of CaMKIV. Their finding showed that *O*-GlcNAcylation keeps CaMKIV in an inactive state and for it to be activated, CaMKIV has to be phosphorylated at a key regulatory site (Thr-200) close to one of the main *O*-GlcNAc sites (S189) after first being de-*O*-GlcNAcylated [78] (Figure 2). Additional findings have shown that OGT is activated by active CAMKIV phosphorylating it [79]. It is unclear if similar mechanisms will be found for other kinases; however, this highlights the extensiveness of this dynamic crosstalk.

## 3. Effects of *O*-GlcNAcylation and Phosphorylation Crosstalk on Regulatory Proteins Expressed by VSMCs

VSMC encompasses an increasingly diverse group of cells which can alter their phenotypic properties in diseases. The understanding and significance of phenotypic switching of VSMC in vascular disease have been aided by recent developments in single-cell transcriptome and lineage-tracing tools [80]. For example, calcium-phosphate metabolism disorders in individuals with chronic kidney disease cause contractile VSMCs to transform into osteoblast-like VSMCs, which leads to vascular calcification [81]. Furthermore, following arterial injury, neointima development is facilitated by synthetic/secretory VSMCs [82]. Also, OxLDL and other molecules that contribute to the lipid-rich necrotic cores of atherosclerotic plaques can be engulfed by macrophage-like VSMCs [83]. These subtypes of VSMCs expresses a repertoire of proteins that are targets for PTM by both *O*-GlcNAcylation and phosphorylation [35,84]. *O*-GlcNAcylation and phosphorylation are similar in that they both modify serine and threonine residues and are dynamically added and removed in response to cellular signals [46]. Mapping the locations where attachments are happening at the same time has proven to be a significant challenge bearing that this knowledge would help predict how the crosstalk will affect cellular response [85]. However, advances in electron capture dissociation and electron transfer dissociation have unlocked new possibilities for mapping *O*-GlcNAcylation and phosphorylation sites [31]. Advances in the characterisation of this crosstalk and possible effects in VSMCs are summarised in Table 1.

## 4. Exploring the *O*-GlcNAcylation and Phosphorylation Crosstalk in VSMCs for Therapeutic Gains in CVDs

*O*-GlcNAcylation presents a conundrum in that, while the sugar alteration is beneficial under acute stress, allowing cells including VSMCs to survive, it causes aberrant signalling, transcription, and cell destruction over time in chronic hyperglycaemia [35,113]. Additionally, the therapeutic significance of its crosstalk with phosphorylation has been elusive. However, convincing evidence shows that *O*-GlcNAcylation and phosphorylation have a substantial interaction, with one modification controlling the cycle enzymes of the other and the two competing for the same site occupancy or at proximal/distal sites on polypeptides [35]. This dynamic crosstalk often serves as a nutrient/stress sensor to regulate transcription, translation, signalling, and the cytoskeleton, which have been linked with altered vascular integrity and the aetiology of chronic CVDs such as atherosclerosis and coronary artery disease [35].

Numerous proteins expressed by VSMCs that regulate vascular tone and cardiac contractility such as PKC, PLC, and P13K are all targets for both *O*-GlcNAcylation and phosphorylation [114,115,116] (Table 1). For example, Hardy et al. have demonstrated that increased *O*-GlcNAcylation of PKC augments vasoconstriction of rat’s aorta as it caused a 47% increase in contractile response to phenylephrine [117]. They further showed that PKC activity increases by 139% when rat aorta *O*-GlcNAc is selectively increased via phosphorylation of PKC-specific myristoylated alanine-rich C kinase substrate (MARCKS) [117]. Additionally, GF109203X, a selective inhibitor of conventional Ca^2+^-dependent PKC isoforms, was shown to prevent PUGNAc-induced contraction of rat aorta [117]. This finding suggested that *O*-GlcNAc augments vasoconstriction by modifying PKC. In another study, treatment of aorta from male Sprague Dawley rats and C57BL/6 mice with PUGNAc caused increased vascular reactivity to vasoconstrictor stimuli phenylephrine and serotonin [118]. Following this observation, it was noticed that there was decreased levels of active phosphorylated endothelial nitric oxide synthase (eNOS) on Ser-1177 and Akt on Ser-473 [118]. These findings [117,118] suggest that *O*-GlcNAcylation augments vasoconstriction responses and that crosstalk with phosphorylation could be a crucial regulatory mechanism in the vasculature.

Additionally, arterial hypertension has been linked to vascular dysfunction arising from increased *O*-GlcNAcylation in aorta of deoxycorticosterone acetate and salt (DOCA-salt) rats [119]. In this study [119], DOCA rat aorta demonstrated reduced vasodilatory response to acetylcholine, and while the contractile response to phenylephrine was significantly greater than aorta from uninephrectomised rats (rats with one kidney excised). Additionally, aorta from the DOCA rats expressed reduced levels of Ser-1177-phosphorylated-activated eNOS and Ser-473-phosphorylated-activated Akt [119]. This study highlighted that *O*-GlcNAcylated proteins are increased in DOCA-salt hypertension, which makes it a target in the treatment of mineralocorticoid hypertension. Additionally, Rigsby et al. [120] have also demonstrated that there is increased protein *O*-GlcNAcylation in renal arteries from angiotensin II/salt-hypertensive rats.

The effect of *O*-GlcNAcylation and phosphorylation crosstalk on FOXP transcription factors such FOXP1 in VSMCs is not well known. The FOX proteins are essential for controlling the transcription of genes linked to the angiogenesis and cell division and development [121]. It has been previously shown that FOXP1 modulates collagen synthesis and proliferation of VSMC by acting as a downstream target of transforming growth factor (TGF)-β [122]. Additionally, it has been demonstrated that downregulation of FOXP1 expression by miR-206 resulted in reduced proliferation, decreased viability, and increased apoptosis of human aortic VSMCs [123]. These findings [122,123] underscore the role of FOXP1 in VSMC viability and proliferation, both of which are essential for vascular restenosis. Therefore, these observations together with the finding of Zhu et al. [65] showing that *O*-GlcNAcylation inhibits FOXP1 activation, suggest that this crosstalk could be explored for therapeutic gains in atherosclerosis and vascular restenosis. Also, we have highlighted in one of our reviews the role of *O*-GlcNAcylation in vascular restenosis and the formation of neointimal hyperplasia that are implicated in VGF [7]. This continues to be a significant problem for patients having coronary artery bypass grafts with saphenous veins as the conduit vessel; it has been estimated that 50% of people without type 2 diabetes mellitus (T2DM) and 80% of patients with T2DM will fail after ten years [7,124,125]. Although the exact impact of *O*-GlcNAcylation and phosphorylation crosstalk in the *O*-GlcNAcylation-driven vascular dysfunction is not fully understood, a growing body of evidence has strongly suggested the link making it a viable target in improving vein graft patency [1,5,7,124,125,126].

**Figure 3:**
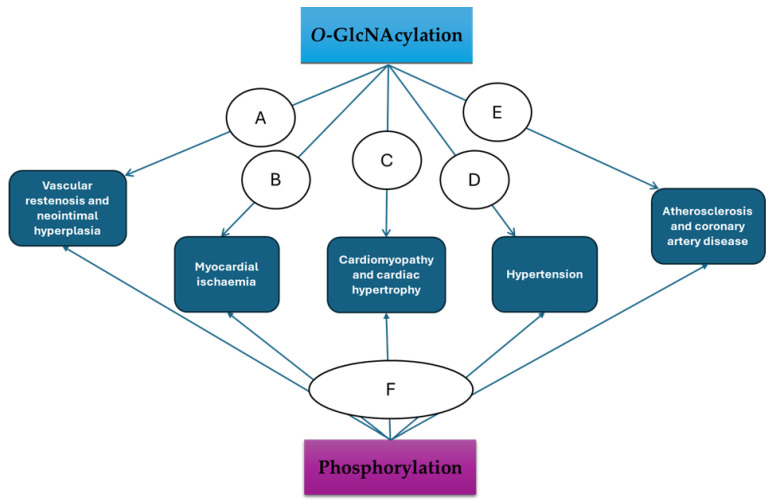
Schematic of key mechanisms by which *O*-GlcNAcylation and phosphorylation crosstalk mediates cardiac and vascular pathologies. **A**: *O*-GlcNAcylation inhibits eNOS activity and promotes VSMC migration and proliferation resulting in vascular restenosis and formation of neointimal hyperplasia [7]. *O*-GlcNAcylation also causes excessive generation of reactive oxygen species (ROS) through NADPH oxidase activation [133]. ROS modulates the activities of miR-200 family of microRNAs [134]. microRNAs play essential role in modulating vascular reactivity [135]. **B**: Acute increases in *O*-GlcNAcylation in response to stress have been shown to enhance cell survival and cardio protection [136]. Conversely, in chronic conditions, it inhibits the activity of acetaldehyde dehydrogenase 2 (ALDH2), an important cardioprotective enzyme, which results in exacerbation of myocardial injury [137]. **C**: *O*-GlcNAcylation upregulates the expression of extracellular signal-regulated kinase 1/2 (ERK1/2), known mediators of cardiac overload causing cardiac hypertrophy [138]. **D**: The IL-10 signalling pathway is inhibited by increased *O*-GlcNAcylation, causing increase in vascular resistance resulting in hypertension. IL-10 is a pleiotropic cytokine that reduces vascular responses to the potent vasoconstrictor endothelin-1 (ET-1) by downregulating the activity of ERK1/2 [139]. **E**: *O*-GlcNAcylation promotes proatherogenic genes like thrombospondin and reduces the action of atheroprotective proteins like eNOS, which in turn promotes atherosclerosis and coronary artery disease [140]. **F**: In response to cellular signal(s), phosphorylation modifies the serine and threonine residues of target proteins to largely limit the effect(s) of *O*-GlcNAcylation.

Targeting *O*-GlcNAcylation as a therapeutic strategy in the management of CVDs remains underexplored and targeting its crosstalk with phosphorylation is more elusive. This is because its involvement in the pathogenesis of CVDs is only now becoming clear. Figure 3 summarises key mechanisms through which *O*-GlcNAcylation and phosphorylation mediate these cardiac and vascular pathologies. The body of evidence described in this review further highlights both the cellular and therapeutic significance of *O*-GlcNAcylation and its crosstalk with phosphorylation in the pathogenesis of CVDs. This presents a strong argument why it should be targeted as treatment strategy in the treatment of CVDs. While the development of OGA inhibitors might be useful for experimental and rare clinical purposes, of interest will be the development of therapeutically effective OGT inhibitors. A substantial amount of data indicates that elevated *O*-GlcNAcylation levels are responsible for changes in the target proteins’ cellular functions that modify vascular and the cardiovascular functions, both of which are linked to CVDs [127]. OGT inhibitors such as alloxan [128] and benzoxazolinones [129] are currently available, but their use has been restricted to in vitro and experimental research as their clinical applications are compromised by their many off-target effects and associated toxicities. However, more recently developed OGT inhibitors like Ac-5SGlcNAc, OSMI 1−4, and L01 may show greater promise as possible pharmacological agents because of their enhanced specificity and favourable pharmacokinetic profiles [130,131,132]. One of our reviews provides a comprehensive analysis of these potential OGT inhibitors [5].

## 5. Conclusions

Over the years, the cellular and therapeutic significance of the *O*-GlcNAcylation and phosphorylation crosstalk has been unclear. However, a plethora of evidence are now emerging highlighting that this dynamic crosstalk plays critical role in several cardiac and vascular pathologies. We have also discovered that the effect of this dynamic crosstalk is cell specific. We have, therefore, through this review, highlighted the cellular and therapeutic significance of this crosstalk in cardiac and vascular pathologies with a focus on VSMCs, a critical cell type that is involved in blood pressure regulation, vascular tone regulation, and vascular contraction and dilatation. We propose that targeting this crosstalk offers a viable therapeutic strategy to treat CVDs.

## Figures and Tables

**Table 1:** Summary of the effects of *O*-GlcNAcylation and phosphorylation crosstalk on VSMC proteins involved in cardiovascular diseases.

Protein	Cardiovascular Function	Effects of Increased *O*-GlcNAcylation	Effects of Phosphorylation	Net Effects of *O*-GlcNAcylation and Phosphorylation Crosstalk	References
Sp1	Transcription factor involved in the regulation of vascular calcification, endothelial dysfunction, fibrosis, and regulation of gene expression	Enhanced activity	Activation	Decreased activity	[59,61,86,87,88]
NF-κB	Regulation of inflammatory response and proliferation of VSMCs	Enhanced activity	Activation	Decreased activity	[89,90,91]
p53	Modulation of metabolism, cell cycle arrest, pro-programmed cell death, and anti-angiogenesis	Enhanced activity	Activation	Decreased activity	[92,93,94]
SERCA2	Regulation of calcium homeostasis	Reduced expression	Enhanced activity	Unknown	[59,95,96]
TGF-β	Vascular remodelling and regulation of the renal renin-angiotensin system	Increased expression	Activation	Decreased activity	[97,98,99]
PKCα,ε	Regulator of cardiac contractility and vascular tone	Altered translocation and expression	Activation	Decreased activity	[74,100]
Akt	Regulator of growth, proliferation, migration, and metabolism of vascular cells	Decreased response to agonists such as insulin	Activation	Decreased activity	[101,102,103]
PI3K	Regulator of cardiac and vascular contractility and growth	Decreased activity and phosphorylation	Activation	Decreased activity	[101,104,105,106]
eIF-2	mRNA translation and regulation	Reduced function	Activation	Decreased activity	[107,108,109]
MAPKs (p38 and ERK1/2)	Regulations of contraction, migration, adhesion, collagen deposition, cell proliferation, differentiation, and survival of vascular smooth muscle cells	Enhance activity	Activation	Enhanced activity	[110,111,112]

## Abbreviations

The following abbreviations are used in this manuscript:

ALDH2	Acetaldehyde dehydrogenase 2
Ca^2+^/CAM-PK	Calcium-calmodulin protein kinase
CaMKIV	Calcium/calmodulin-dependent protein kinase IV
CHK1	Checkpoint kinase 1
CVD	Cardiovascular disease
DOCA	Deoxycorticosterone acetate
eIF-2	Eukaryotic initiation factor-2
eNOS	Endothelial nitric oxide synthase
ERK1/2	Extracellular signal-regulated kinase 1/2
FOXP1	Forkhead box protein P1
GFAT	Glutamine-fructose-6P amidotransferase
GlcN-6-P	Glucosamine-6-phosphate
GNPNAT	Glucosamine-6-phosphate *N*-acetyltransferase
GSK-3	Glycogen synthase kinase-3
MAPKs	Mitogen-activated protein kinase
NF-κB	Nuclear factor kappaB
OGA	*O*-GlcNAcase
*O*-GlcNAc	β-D-*N*-acetylglucosamine
OGT	*O*-GlcNAc transferase
PGM3	GlcNAc phosphomutase
PI3K	Phosphoinositide 3-kinases
PKC	Protein kinase C
PKG	cGMP-dependent protein kinase
PLC-β1	Phospholipase C-beta 1
PTKs	Tyrosine protein kinases
PTM	Post-translational modification
PUGNAc	O-(2-acetamido-2-deoxy-d-glucopyranosylidene)-amino-N-phenylcarbamate
SERCA2	Sarco/endoplasmic reticulum calcium (Ca^2+^)-ATPase
Sp1	Specificity protein 1
T2DM	Type 2 diabetes mellitus
TGF-β	Transforming growth factor beta
UAP/AGX1	UDP-*N*-acetylhexosamine pyrophosphorylase 1
UDP-GlcNAc	Uridine diphosphate-*N*-acetylglucosamine
VSMCs	Vascular smooth muscle cells
